# Genomic Research to Identify Novel Pathways in the Development of Abdominal Aortic Aneurysm

**DOI:** 10.1155/2012/852829

**Published:** 2012-01-29

**Authors:** Seamus C. Harrison, Anastasia Z. Kalea, Michael V. Holmes, Obi Agu, Steve E. Humphries

**Affiliations:** ^1^Centre for Cardiovascular Genetics, BHF Laboratories,The Rayne Building, Department of Medicine, University College London (UCL), 5 University Street, London WC1E 6JF, UK; ^2^Genetic Epidemiology Group, Department of Epidemiology and Public Health, University College London, 1-19 Torrington Place, London WC1E 7HB, UK; ^3^Department of Vascular Surgery, University College London Hospital, London NW1 2BU, UK

## Abstract

Abdominal aortic aneurysm (AAA) is a common disease with a large heritable component. There is a need to improve our understanding of AAA pathogenesis in order to develop novel treatment paradigms. Genomewide association studies have revolutionized research into the genetic variants that underpin the development of many complex diseases including AAA. This article reviews the progress that has been made to date in this regard, including mechanisms by which loci identified by GWAS may contribute to the development of AAA. It also highlights potential post-GWAS analytical strategies to improve our understanding of the disease further.

## 1. Introduction

Abdominal aortic aneurysm (AAA) is a common, late onset disease which, left untreated, can rupture with a high resultant mortality. Approximately 5% of Caucasian males aged 65–74 will harbor a AAA [1] and the major risk factors for the condition include male sex, cigarette smoking, a history of cardiovascular disease, and a family history of AAA [2, 3]. Currently, the best predictor of rupture is maximal aneurysm diameter and surgical repair is indicated in AAA greater than 5.5 cm [4]. Population screening with abdominal ultrasound scans (US) reduces the burden of aneurysm related death [5, 6], but there is a lack of evidence to support any pharmacological therapies to attenuate AAA progression and/or rupture. The advent of endovascular aneurysm repair has reduced short-term perioperative mortality associated with AAA repair [7] but nationwide audits indicate that elective repair still carries a mortality risk in region of 1.5–7% [8]. In patients deemed unfit for surgical repair ten-year survival is less than 25% [9]. Understanding the genetic architecture of the condition may provide a blueprint for uncovering novel pathobiological pathways and targets for nonsurgical treatments.

The role that genetic factors play in the development of AAA has become increasingly prominent in recent years following Clifton's initial observation that the disease appeared to run in families [10]. Family history of AAA is an established risk factor for the disease, with male first-degree relatives of probands at approximately fourfold greater risk than the general population [11–13]. A twin-study of AAA has estimated the heritability to be as high as 70% [14], and familial studies have failed to demonstrate consistent modes of inheritance, suggesting that it is likely to be a complex disease [13, 15], resulting from a complicated network of environmental and genetic risk factors. There has been some progress in discovery of rare monogenic cause of aneurysmal disease in thoracic aorta (Table 1) but in common with other complex disorders, deciphering causal genetic variants in AAA has proved a difficult task. Familial-based linkage studies have identified areas of the genome that are strongly associated with the disease, but attempts to refine the signal have so far been unsuccessful [15, 16]. 

## 2. Genetic Studies of AAA

### 2.1. Candidate Gene Approaches

The “common-disease common-variant” hypothesis poses that common complex diseases arise from the accumulation of genetic variants, each with a modest effect on risk (low penetrance) and environmental risk factors [22, 23]. It is this hypothesis that has underpinned the developments of genetic association studies, whereby the frequency of indexed genetic variants is compared between cases and controls.

A number of candidate gene association studies for AAA have been published. Review of the literature, however, reveals that many studies were underpowered and gave inconsistent results, a problem shared by many other complex disorders [24]. Small studies with a low *P* value obtained by chance have been more readily published than negative findings (so-called publication bias), and the results are often not replicated in larger studies with greater statistical power. Despite this, meta-analysis of candidate gene studies suggests that single nucleotide polymorphisms (SNPs) in genes of the renin-angiotensin system and folate metabolism are consistently associated with an increased risk of developing AAA (Table 2) [25, 26]. There has been considerable interest in the role of polymorphisms in the TGF-*β* superfamily and risk of developing AAA as these genes have been causally implicated in aneurysmal disease affecting the thoracic aorta. Baas et al. found association between SNPs in TGF-*β* receptor 1 and 2 (*TGFBR1 *and* TGFBR2*) and risk of AAA in a Dutch cohort [27], but these associations were not replicated in two cohorts from New Zealand and Australia [28]. There have also been studies demonstrating suggestive associations between SNPs in Latent Transforming Growth Factor 4 (*LTBP4*) and expansion of AAAs, but again, this finding has not been replicated in independent follow-up studies [29]. 

Efforts by the Human HapMap consortium (http://hapmap.ncbi.nlm.nih.gov/index.html.en/), the SNP consortium (http://www.ncbi.nlm.nih.gov/SNP/), and more recently the 1000 genome project (http://www.1000genomes.org/) have uncovered much of the common variation seen throughout the human genome. Linkage disequilibrium (LD: the nonrandom association of alleles at two or more loci) means that only a fraction of all possible SNPs require genotyping, in order to impute information on nontyped genetic variation, and chips that simultaneously genotype up to 1 million variants at a time are commercially available.

In genomewide association studies (GWASs), a panel of common SNPs (minor allele frequency >5%) capturing common genetic variation across the entire genome is compared in groups of cases and controls. This approach is “hypothesis-free” and therefore not subject to potential biases seen in candidate gene studies. Owing to the large number of independent test in a single association study, there are many factors to consider when designing a GWAS. Most importantly, this multiple testing strategy results in a large number of potentially false positive associations. To adjust for this, stringent criteria for “genome wide significance” are applied and replication of findings in independent cohorts is required. A potential consequence of this is that many true-positive associations may be lost in the “statistical noise”. A second issue is that with only a few exceptions, the effect size of common variants is small. Carriers of risk alleles are generally at 10–30% increased odds of disease compared to noncarriers. These characteristics necessitate extremely large sample sizes in order to have sufficient statistical power, with recent publications combining multiple studying hundreds of thousands of subjects at a time [14, 30]. As of June 2011, 951 GWASs have now been published in a wide range of disorders and traits (http://www.genome.gov/gwastudies/).

### 2.2. GWASs and AAA

In 2007, the field of genomic research was ignited by simultaneous publication of three GWASs of cardiovascular disease [31–33]. Each of the studies demonstrated a strong association between common SNPs on Chromosome 9p21.3 [34], in a gene desert (an area of the genome with no known protein-coding genes). These data exemplified the power of GWAS, as this locus would not have been given priority using a candidate gene approach. The limitations were, however, also highlighted as the functional significance of this locus was unclear. It has taken a further 3-4 years to understand the biological mechanisms by which these variants act, and the translational benefit of these discoveries is not likely to be realized in the near future. A year following publication of these GWAS, it was reported that SNPs at this locus were also strongly associated with the presence of AAA [34]. These SNPs are common in the population (risk allele frequency ~45–50%), and individuals carry 0, 1, or 2 risk alleles. The risk of developing AAA is increased by ~30% per allele carried. The association with AAA has now been replicated in a number of well-powered case-control studies (Table 3) [35–37].

The first GWAS specifically of AAA was published in 2009 and identified association of on SNP on Chr3p12.3 with AAA [38]. This association did not meet conventional levels of genomewide significance and has not been replicated in independent sample sets [39]. However, in 2010 a larger GWAS with greater statistical power reported a novel association with sequence variant in *DAB2IP* on Chr9q33 [40]. The discovery phase included 1,292 individuals with AAA (defined as an infrarenal aortic diameter >3 cm) and 30,530 unscreened controls (a small proportion of whom are likely to harbor AAA), while follow-up replication studies included 3,297 cases and 7,451 controls (all cases and controls were of European ancestry). The variant conferred a per allele odds ratio for AAA of 1.21, a smaller effect than that seen with the 9p21 variant. Interestingly, the investigators also found an association between this SNP and CHD, venous thromboembolism, and peripheral arterial disease and the association with CHD has now been replicated in further independent cohorts [41]. Further GWASs are expected in the future [30], and it is possible that meta-analyses of these datasets will uncover further variants associated with the disease.

### 2.3. Functional Analysis of GWAS Loci to Uncover Novel Pathobiological Pathways

Initial excitement from three separate GWAS reporting robust associations between common risk variants on Chr9p21.3 and myocardial infarction was tempered by the fact that the functional significance of the locus was not immediately obvious. The lead SNP (or any in close LD with it) does not lie in a protein coding gene. It has, however, been identified that this risk variant overlaps with the recently annotated noncoding RNA (ncRNA), *ANRIL.* NcRNAs can alter expression of protein coding genes by mechanisms such as gene silencing, DNA methylation, chromatin remodeling, and RNA interference [42]. Functional studies of this locus have demonstrated that carriers of the risk variant have reduced expression of *ANRIL*, along with other nearby genes such as *CDKN2A *and *CDKN2B *[43] which are inhibitors of cellular senescence involved on controlling cellular proliferation and apoptosis. Jarinova et al. found that the risk locus has enhancer activity in primary human aortic smooth muscle cells and that pathways involved in cellular proliferation were upregulated in risk allele carriers [44]. Visel et al. then demonstrated that targeted deletion of this region in a mouse model leads to increased expression of the *CDKN2A *and *CDKN2B *and that aortic smooth muscle cells from these animals displayed excessive proliferation and diminished senescence [45]. More recently, it was shown that the region at 9p21 is densely packed with enhancer sites that are capable of altering expression of both neighboring and distant genes by physical interaction. Specifically, variants associated with CHD (and AAA) disrupt binding of the transcription factor STAT1, which results in altered expression *CDKN2A *and *CDKN2B, MTAP *(methylthioadenosine phosphorylase, an enzyme that plays a major role in polyamine metabolism), and *IFNA21 *(interferon alpha-21). It was also demonstrated that the transcriptional control of the 9p21 enhancers was remodeled with interferon-*γ*, providing evidence that genetic variation is one factor that determines the response to inflammatory stimuli within the vasculature [46].

The SNP in *DAB2IP *discovered by GWAS also associates with coronary artery disease, peripheral arterial disease, venous thromboembolism, and pulmonary embolism but shows no association with any classical CHD risk factors [40, 41]. *DAB2IP, *located on Chromosome 9q33, is a GTPase activating protein thought to play an important role in prostate cancer metastasis [47]. A SNP in this gene has been associated with aggressive prostate cancer [48], while *in vitro* functional studies have demonstrated that loss of the protein leads to enhanced cell proliferation and reduced apoptosis, via the PI3-Akt pathway [49]. *DAB2IP* expression is significantly reduced in AAA tissue compared to tissue from healthy controls [50], and this SNP did correlate with reduced expression of the protein in aortic tissue (though this was not reproduced in mammary artery tissue) [40]. It is possible, therefore, that this variant also promotes excessive vascular smooth muscle cell (VSMC) proliferation, through reduced expression of *DAB2IP* in aortic tissue. Interestingly, *DAB2IP *expression is modulated by EZH2, a histone methyltransferase forms part of the polycomb repressor complex, and has been proposed as a potential drug target in prostate cancer [51, 52]. If, at a molecular level, the link between genetic variation at this locus, *DAB2IP *expression, and vascular disease was uncovered, enzymes such as EZH2 could also be potential novel targets in pharmacological therapies to attenuate AAA formation.

Whilst it appears that the two SNPs discovered for AAA may both be influencing a common disease pathway, there was no evidence of epistatic interaction between the 9p21 and *DAB2IP *SNP, with simply additive effects on AAA risk [40]. We have found the same with regard to risk of CHD; approximately 40% of the population who carry 2 or more risk alleles at these loci have a hazard ratio for myocardial infarction of 1.7 compared to individuals carrying zero risk alleles [41]. This suggests that accumulation of small disturbances in different elements of the VSMC proliferation pathway combines to increase the risk of both atherosclerosis and AAA.

The small effect sizes seen with GWAS-identified variants do not preclude potential biological importance, as they may highlight important pathways in disease [53]. For example, genes highlighted by GWAS of type 2 diabetes mellitus (T2DM) are known targets for thiazolidinediones and sulphonylureas [54], drugs commonly used in this condition. For AAA, the genomewide data are pointing to pathways involved in promoting excessive VSMC proliferation. Cigarette smoking, a major environmental risk factor for both diseases, leads to increased levels of proliferation in VSMCs [48, 55], whilst a role for excessive VSMC proliferation in aneurysm formation elsewhere in the arterial tree has been demonstrated—mutations in *ACTA2 *(smooth muscle actin alpha 2) and *TGFBR2* (transforming growth factor beta receptor 2) promote excessive VSMC proliferation and are causal for thoracic aneurismal disease [19, 56]. Indeed, evidence from candidate gene studies also suggests a role for excessive VSMC proliferation. The Angiotensin II type 1 receptor 1166C polymorphism has been associated with AAA in three independent cohorts [26] (per allele odds ratio 1.60, 95% CI 1.32–1.93, *P* = 1.1 × 10^−6^), and it has been shown that this polymorphism increased vascular response to circulating Angiotensin II [57], a potent stimulator of VSMC proliferation and migration [58].

## 3. Future Directions for Genomics and Pathobiology of AAA

### 3.1. Study Design to Refine and Augment Signals

In genetic studies a useful alternative to dichotomizing complex disorders is to consider the clinical end-point as a combination of different continuous traits [59]. Within the population, infrarenal aortic diameter is a continuously distributed phenotype (skewed to the right) [60], with AAA rupture (the clinical end-point of interest) in aortas less than 4 cm almost unheard of. Rather than dichotomizing into AAA versus no AAA (cut-off threshold 3 cm), another option as suggested by Plomin [59] would be to study the trait across the range of variation in the population. This strategy has been used to great effect in other complex disorders; for example, following discovery of loci for T2DM, a binary outcome, signals have been refined by studying continuous traits associated with the disease such as fasting glucose, insulin secretion, and obesity. Population-based studies provide greater freedom from biases, better definition of environmental exposures before disease onset, and clearer characterization of the evolution of traits over time [61].

Another area that has received limited attention in the literature to date is the discovery of variants that associate with rapid aneurysm expansion of small AAA. It is not clear whether this phenotype has a large heritable component or whether the genes that predispose to AAA are also those that predispose to rapid expansion. For example, it does not appear that the 9p21 SNP associates with expansion rates [36]. It should be noted, however, that genetic studies of expansion have often been small and underpowered, with heterogeneity in the cohorts with regard to how the phenotype is actually measured and modeled, which is a major methodological concern. These problems will only be overcome by a large-scale collaborative effort to produce standardized methods for phenotype definitions and data-collection.

### 3.2. Rare Variants/Exome Sequencing

Despite the discovery of large numbers of variants associated with many complex diseases by GWAS, the majority of observed heritability in most of these diseases remains unexplained [62]. This has prompted speculation that rare variants of large effect (which are poorly covered on currently available gene chips) may be important in development of common diseases [62]. Rare causative mutations have been identified by exome-sequencing experiments in a handful of single genedisorders [63, 64], whilst deep resequencing efforts have identified rare variants of large effect at loci implicated by GWAS in traits such as triglyceride levels [65]. Despite these successes, the whole genome/exome resequencing studies for common complex diseases remain limited by expense, statistical power, and computational capacity [66].

### 3.3. MicroRNAs and AAA

MicroRNAs (miRNAs) are a class of endogenous noncoding single-stranded RNAs (19–24 nucleotides) that are important regulators of gene expression. miRNAs are transcribed as primary miRNAs (pri-miRNAs), processed to precursor miRNAs (pre-miRNAs), and then to mature miRNAs. It is estimated that more than 60% of protein-coding genes are regulated by these small RNAs [67, 68], composing a new complicated regulatory network with a significant role in biological functions that are frequently regulated cooperatively by large numbers of genes.

The VSMC is crucial to the progression of almost all vascular wall disorders including AAA [69, 70]. Liu et al. studied the expression of miRNAs in an experimental animal model of AAA and discovered a group of miRNAs differentially expressed in AAA versus normal Sprague Dawley rat aortas [71]. Bioinformatics analyses for predicted mRNA targets of differentially expressed miRNAs showed enrichment for cell signaling pathways thought to play a role in human AAA development, such as the mitogen-activated protein kinase pathway. Recently Leeper et al. performed an *in vitro* miRNA microarray analysis of human VSMC to identify 28-upregulated and 3-downregulated miRNAs that were significantly and sustainably altered during the differentiation process [72]. Among the regulatory miRNAs for VSMC, miRNA-26a was of particular interest as it appeared to serve as an inhibitor of VSMC differentiation by inhibiting the effect on the signaling pathways downstream of the TGF-*β*/BMP superfamily of growth factors. Cells deficient in miRNA-26a lost their migratory phenotype toward a growth factor/serum gradient and displayed enhanced rates of programmed cell death. These effects on the TGF-*β* pathway and on VSMC proliferation, migration, and apoptosis in particular suggested that miRNA-26a could be important in AAA development. These results were confirmed using two independent animal models of AAA disease, where miRNA-26a expression coincides temporally with VSMC apoptosis and cell loss.

**Figure 1:**
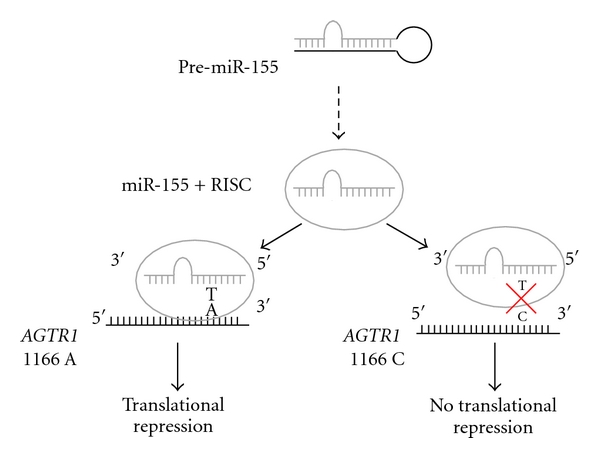
Model of miR-155 binding to the 3′UTR of *AGTR1* and association with the 1166A>C polymorphism (rs5186). The hairpin-shaped precursor of miR-155 (pre-miR-155) after being exported in the cytoplasm is assembled into the RNA-induced silencing (RISC) complex (mature miR-155), which transfers it to recognise specific mRNA targets. miR-155 binds with complete sequence complementarity to its target mRNA seed site and induces posttranscriptional gene silencing. In the presence of the −1166 C allele in the 3′UTR of *AGTR1, *the compensatory base pairing is affected, and miR-155 binding is inhibited leading to increased *AGTR1* expression.

Another miRNA-related mechanism of diseases susceptibility is where SNPs alter miRNA target sites. For example, the 1166A>C polymorphism (rs5186) in the angiotensin receptor 1 (*AGTR1*) that has been associated with AAA by candidate gene analysis appears to abolish miR-155-mediated regulation of the AGTR1 gene [73, 74] (Figure 1). Angiotensin II receptor type 1 (AT1) signaling stimulates proliferation of VSMC and vascular fibrosis [75], while the AT1 receptor blocker Losartan has been shown to reduce experimental aneurysm formation in mouse models of Marfan's disease [76]. Daugherty et al. showed that selective blockade of AT1 signaling with Losartan attenuated AAA formation in the Angiotensin II-infused apoE−/− mouse model, but blockade of AT2 signalling resulted in more severe atherosclerosis and aneurysmal disease [77]. Taken together, these data suggest that micro-RNAs are likely to play a role in the remodeling process seen during aneurysm development, and further research of genetic variation in both microRNAs and their targets may uncover some novel insights. 

## 4. Conclusions

Whilst GWASs are redefining our understanding of many complex diseases including AAA, it is clear that they represent only an early step in the process of genetic discovery. It is too early to define specific translational roles for any of the loci identified so far that associate with AAA, but light is being shed on pathobiological pathways such as those involved in excessive VSMC proliferation, which has potential implications for development of nonsurgical therapies. Further discoveries will rely upon collaboration of large research consortia as seen in other complex diseases and careful consideration of how information from genomewide data could be harnessed to develop specific therapies and individualized preventative strategies.

## Figures and Tables

**Table 1:** Monogenic causes of thoracic aortic diseases.

Phenotype/syndrome	Gene	Reference
Marfan syndrome	*FBN1*	[17]
Loeys-Doetz—ascending aortic aneurysm	TGFBR1 and TGFBR2	[18]
Thoracic aortic aneurysm	MYH11, ACTA2, SMAD3	[19–21]

**Table 2:** SNPs associated with AAA after meta-analysis of candidate gene studies [25, 26].

Gene/polymorphism	Number of studies (total cases/controls)	Effect size (OR and 95% CI)
Angiotensin type 1 Receptor/A116C (rs5186)	1 study, 3 populations (1226/1712)	1.386 (1.2–1.601)
Angiotensin converting Enzyme I/D (rs4646994)	4 (1657/2238)	1.238 (1.12–1.36)
Methlyenetetrahydrofolate reductase +677C>T	5 (1086/895)	1.234 (1.020–1.494)
Matrix metalloproteinase 9 (MMP9, 1562C>T)	3 (848/802)	1.09 (1.01–1.18)

**Table 3:** Association with SNPs in the 9p21 locus with AAA.

Author	Cases/Controls	SNP	OR (*P*-value)
Helgadottir et al. [34]	2836/16732	rs10757278	1.31 (1.2*E* − 12)
Bown et al. [35]	899/815	rs1333049	1.22 (0.004)
Thompson et al. [36]	741/1366	rs10757278	1.38 (0.03)
